# Sol-Gel Deposition of Iridium Oxide for Biomedical Micro-Devices

**DOI:** 10.3390/s150204212

**Published:** 2015-02-12

**Authors:** Cuong M. Nguyen, Smitha Rao, Xuesong Yang, Souvik Dubey, Jeffrey Mays, Hung Cao, Jung-Chih Chiao

**Affiliations:** 1 Department of Electrical Engineering, University of Texas, Arlington, TX 76019, USA; E-Mails: smitha@uta.edu (S.R.); xuesong.yang@mavs.uta.edu (X.Y.); souvik.dubey@mavs.uta.edu (S.D.); jeffrey.mays@mavs.uta.edu (J.M.); jcchiao@uta.edu (J.-C.C.); 2 Department of Electrical Engineering, ETS, Montreal, QC H3C 1K3, Canada; E-Mail: Hung.Cao@lacime.etsmtl.ca

**Keywords:** dopamine, Nernst-response, neurotransmitter sensors, pseudo-reference electrodes, sol-gel IrO_x_

## Abstract

Flexible iridium oxide (IrO_x_)-based micro-electrodes were fabricated on flexible polyimide substrates using a sol-gel deposition process for utilization as integrated pseudo-reference electrodes for bio-electrochemical sensing applications. The fabrication method yields reliable miniature on-probe IrO_x_ electrodes with long lifetime, high stability and repeatability. Such sensors can be used for long-term measurements. Various dimensions of sol-gel iridium oxide electrodes including 1 mm × 1 mm, 500 μm × 500 μm, and 100 μm × 100 μm were fabricated. Sensor longevity and pH dependence were investigated by immersing the electrodes in hydrochloric acid, fetal bovine serum (FBS), and sodium hydroxide solutions for 30 days. Less pH dependent responses, compared to IrO_x_ electrodes fabricated by electrochemical deposition processes, were measured at 58.8 ± 0.4 mV/pH, 53.8 ± 1.3 mV/pH and 48 ± 0.6 mV/pH, respectively. The on-probe IrO_x_ pseudo-reference electrodes were utilized for dopamine sensing. The baseline responses of the sensors were higher than the one using an external Ag/AgCl reference electrode. Using IrO_x_ reference electrodes integrated on the same probe with working electrodes eliminated the use of cytotoxic Ag/AgCl reference electrode without loss in sensitivity. This enables employing such sensors in long-term recording of concentrations of neurotransmitters in central nervous systems of animals and humans.

## 1. Introduction

Implantable electrochemical sensors have emerged as one of the more attractive means for *in situ* sensing and monitoring biological conditions such as pH, pO_2_, and pCO_2_, physiological signals such as ECG, EEG, and biological analytes such as glucose, lactate, uric acid and neurotransmitters [1–7]. There is a growing interest in miniaturizing the sensors for long-term recording. Several widely acknowledged advantages of smaller sensors include minimization of local tissue damage, reduction of inflammation, and improvement of large-scale integration and spatial resolution [3,4,8]. Advancements in microelectromechanical system (MEMS) and micromachining technologies have played a substantial role in the quest for miniaturizing implantable sensors with batch production. Recent fabrication technologies also allow integrating multiple microelectrode arrays (MEAs) on different substrates such as silicon, ceramic, glass and polyimide [5,9–13].

Reference electrodes (RE), the mandatory parts of the electrochemical sensors, are important to provide reference points for measurement [14–16]. Most biochemical sensors utilize dedicated silver/silver chloride (Ag/AgCl) wires as the REs in *in vivo* experiments [3,17]. The Ag/AgCl wires are normally placed apart with a superfluous distance from the sensing sites due to difficulties of implantation processes [17]. Thus, sufficient ionic contact between the Ag/AgCl wires and the working electrode (WE) is required to guarantee a valid measurement of the sensor. Additionally, the Ag/AgCl RE is inconvenient, for example, in long-term experiments in which the sensor might be anchored to a mammalian organ and the additional electrodes would induce more injuries to tissues. Excessive noises are also predicted with the distant RE. Therefore, it is compelling that MEAs integrate both reference and working electrodes on the same probe [17].

On-probe planar Ag/AgCl reference electrodes have been considered in our previous work [7,18]. However, many issues have been identified in biological applications. First, the traditional laboratory fabrication method using electroplating of bulk silver in a saturated chlorine solution has low throughput and is unrepeatable [19]. Second, the fabricated Ag/AgCl films easily delaminate and dissolve in long-term experiments, especially in biological environments with less chloride ions [16,19–21]. Third, bulk and nano-structured silver electrodes have demonstrated cytotoxicity to living tissues in both acute and long-term experiments [22,23]. Iridium oxide (IrO_x_)-based electrodes have emerged as an attractive alternative for REs due to their unique properties. IrO_x_ has been widely accepted in neuroscience applications, especially electrophysiological recording and stimulation because of its high charge density, biocompatibility, and corrosion resistance in electrolyte solutions [24–27]. In addition, IrO_x_ electrodes, owing to their stable mechanical properties on both rigid and flexible electrodes, have been promisingly used for long-term experiments [17]. Although the electrode potential of IrO_x_ is pH-dependent, it has been proven that the potential varies within a small dynamic range in biological environments where the change is less than 0.8 pH units [17,28,29]. Thus, we proposed to utilize IrO_x_ electrodes as reference electrodes for biomedical recording; and we refer to them as pseudo-reference electrodes [30].

IrO_x_ could be fabricated by different methods, such as directly sputtering using an IrO_x_ target, thermal oxidation of iridium, anodic electrodeposition of IrO_x_, and sol-gel dip-coating. Sputtering and thermal oxidation of iridium are not preferable due to cost of materials needed. Electrodeposition has been well-developed and allows depositing hydrated IrO_x_ onto micro-scale electrodes [17,31]. However, the main shortcomings of the electrodeposited IrO_x_ films are low adhesion to their substrates and delamination in biological environments [32,33]. In addition, an issue of over-electrodeposition yielding conductive IrO_x_ dendrites outside of the electrode areas could consequently cause cross-talk between electrodes in the MEA. Finally, higher dependence of such hydrated IrO_x_ to pH change endowing a super-Nernst response of 66–72 mV/pH [33] is not desirable for pseudo-REs.

Sol-gel dip-coating has been implemented to fabricate IrO_x_ electrodes for miniature pH sensor arrays [8,18,34]. The method yields anhydrous IrO_x_ films exhibiting the Nernst response around 56 mV/pH, which is lower than that of the electrodeposited IrO_x_ [8,18]. Additionally, the IrO_x_ film becomes stable and highly adhesive to the substrates after heat-treatment [18]. In this work, we investigated the implementation of the sol-gel-based IrO_x_ films for pseudo-REs. The previous fabrication method introduced a thick SU-8 photoresist mask to precisely and selectively coat the gel solution on pre-defined electrodes [18]. However, the sacrificial layers degraded and became hard to remove after the heat treatment at 300 °C, thus it was difficult to apply the process for small electrode sites. Utilizing sol-gel processes with two-step heat treatment could prevent the aforementioned problem with the sacrificial layers. While the first step of pre-heating at a low temperature was aimed at dehydrating the sol-gel solution, the second one of curing at higher temperature was applied to yield a more adhesive amorphous IrO_x_ thin film. The sacrificial photoresist mask was stripped off between these two steps. The lower temperature of the dehydration step allowed easy removal of the SU-8. Thus, it became possible to fabricate miniature electrodes with different dimensions on both rigid and flexible substrates.

In this work, we implemented the modified sol-gel deposition to fabricate miniature IrO_x_ electrodes. Various IrO_x_ electrode designs have been fabricated on flexible substrates targeting biological applications. The pH-dependence of these electrodes was investigated. Long-term stability was examined in *in vitro* experiments by immersing the electrodes in acidic, alkaline and neutral solutions. The sol-gel-based IrO_x_ electrode was integrated as the pseudo-RE of a dopamine (DA) sensor. The simple, cost-effective and high-throughput manufacturing processes yielded the DA sensor with many advantages such as high stability, reproducibility and high resolution.

## 2. Materials and Methods

### 2.1. Sensor Fabrication

**Figure 1.**
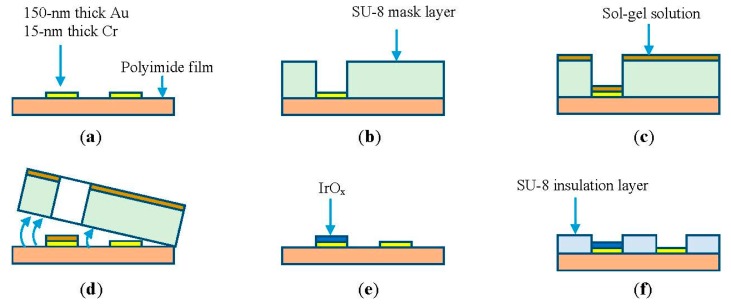
(**a**) Microelectrodes patterned on a flexible polyimide substrate; (**b**) A 50-μm thick SU-8 mask layer was fabricated for sol-gel deposition; (**c**) Sol-gel deposition of iridium chloride solution on the micro-electrodes; (**d**) The first heat treatment step at 100 °C for 15 min was applied prior to peel-off of the mask layers; (**e**) The second heat treatment at 300 °C was applied for four hours to produce IrO_x_; (**f**) A 5-μm thick SU-8 insulation layer was coated on the entire device except the sensing areas and connection pads.

Microelectrodes of different dimensions were fabricated on a 125-μm polyimide flexible substrate. The substrate was cleaned with acetone and rinsed before applying 15-min heat treatment at 100 °C. Photolithography was carried out to pattern the microelectrodes of different sizes including 1 mm × 1 mm, 500 μm × 500 μm, and 100 μm × 100 μm for investigation. This was followed by deposition of layers of 15-nm thick chromium and 150-nm thick gold using an e-beam evaporator, as depicted in Figure 1a. Lift-off was carried out to release sacrificial layers revealing the micro-patterns. Next, the negative-tone photoresist SU-8 (MicroChem, Newton, MA, USA) was spin-coated to form a mask layer. The thicker the SU-8 layer was, the easier it was to peel-off later. However, it was tedious to handle the device during fabrication processes. Thus, the thickness of the sacrificial mask layer was chosen as 50 μm. Different types of SU-8 including SU-8-2025, SU-8-50, and SU-8-100 were tested. Based on our experimental data, we chose SU-8-50 to employ in the 50-μm thick mask, as illustrated in Figure 1b. The sample was then coated with an iridium chloride sol-gel solution (Figure 1c). The preparation of the solution and coating processes are described in Section 2.2. Figure 1d illustrates the peeling-off process after the first heat treatment step. The second heat treatment at 300 °C was carried out to form a thin layer of iridium oxide (Figure 1e). In the final step, a 5-μm thick SU-8-5 layer was coated as an insulation layer covering the entire microelectrode except the sensing areas and connection pads, as shown in Figure 1f. Finally, copper wires from external measurement circuits were connected to the pads using silver epoxy.

### 2.2. Sol-Gel Deposition

The sol-gel solution was mixed according to the recipe given in [8] by dissolving 0.5 g iridium chloride in 21 mL of ethanol (95%, Sigma-Aldrich, St. Louis, MO, USA) and 5 mL of acetic acid solution (99.7%, MG Scientific, Pleasant Prairie, WI, USA). The solution was stirred by a magnetic rod for one hour. Dip-coating process was carried out by immersing the entire substrate in the sol-gel solution. The SU-8 mask exposed only the sensing area of the micro-electrodes to the sol-gel solution for coating. After immersing the electrodes in the sol-gel solution for a few minutes, the whole substrate was drawn out with a withdraw rate of 2 cm/min using a dip-coating apparatus [8,34]. This withdraw rate was experimentally shown to provide good film uniformity. The film quality and thickness showed significant improvement when the dipping process was repeated five times.

**Figure 2.**
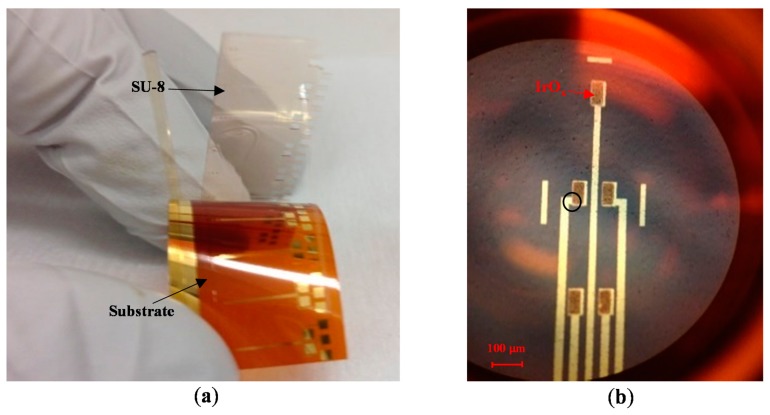
(**a**) Photo of the peeled-off SU-8 mask layer after the first heat treatment; (**b**) A photo of the MEA with the sensing areas coated with IrO_x_. The sensing film size of each electrode was 50 × 100 μm^2^. The black circle on the top-right electrode of the MEA indicates the area imaged by a scanning electron microscopy in Figure 3b.

After the sol-gel dip coating step, the sample was subjected to heat treatment to generate IrO_x_ from iridium chloride. First, a heat treatment step at 100 °C for 15 min dehydrated the sol-gel solution. Afterwards, the SU-8 sacrificial layer was peeled-off by bending the flexible substrate, as shown in Figure 2a. It should be noted that the adhesion of SU-8 to the substrate is poor; therefore, the sacrificial mask was automatically detached by mechanical stresses. Next, a second heat treatment step was carried out with the samples in an oven. The heating profile was: (1) ramp-up to 300 °C at a rate of 2 °C/min; (2) soak at 300 °C for four hours; and then (3) ramp-down to 25 °C at a rate of 1 °C/min. This step completed conversion of hydrated iridium chloride into iridium oxide, as illustrated by color change from brown to blue in Figure 1e. The optical microscope photo in Figure 2b shows an MEA with five electrodes. Each electrode has opening area of 50 × 100 μm^2^ and was coated with IrO_x_.

### 2.3. Material Characterization

**Figure 3.**
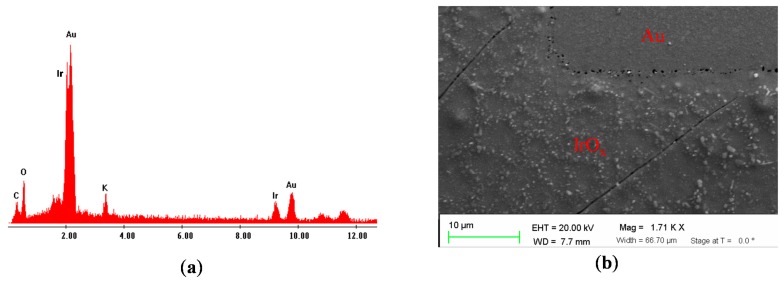
(**a**) Energy dispersive spectroscopy (EDS) shows visible peaks of Ir, Au, C, O, and K; (**b**) Scanning electron microscopic image of the surface on the microelectrode as indicated with the black circle in Figure 2b.

Iridium oxide fabricated by sol-gel deposition was characterized by energy dispersive spectroscopy (EDS). Figure 3a shows the material composition on the surface of the electrode after sol-gel deposition. There were clear peaks of different materials including Ir, Au, C, O and K with composition percentages of 18.19%, 47.18%, 17.69%, 15.19% and 1.74%, respectively. The presence of carbon element was due to polyimide substrate while there was small amount of potassium due to contamination. Scanning electron microscopy was also utilized to examine of the surface morphologies of the micro-electrode. The composition of the film is similar to the one made by one-step sol-gel heat treatment. Figure 3b shows the surface of the electrode coated with IrO_x_, which was rougher than the one in the Au area. Micro-scale porous structures of IrO_x_ were observed. The porous structures with a high surface-to-volume ratio could affect electrochemical properties of the bulk IrO_x_ such as the ion exchange, rate to reach the equilibrium point of reversible reaction, or the amount of charge storage [18].

## 3. Experiments and Results

Typical electrochemical sensors have 3-electrode configuration with a working electrode (WE), a reference electrode (RE) and an auxiliary or counter electrode (CE) [35,36]. Applications using micro-electrochemical sensors could eliminate the CE since output signals of the sensor are small [3,35,36]. Among many electrochemistry methods, amperometric sensors require a fixed bias voltage applied between their WEs and REs. Since the output signals of the miniature amperometric sensors are in the range of picoamps, the signals could be affected by minute changes of the bias voltage. Consequently, one of the important characters of the ideal RE is to provide a constant open-circuit potential regardless of electrolyte solutions. The change of IrO_x_ electrode potential obeys its pH sensitivity. However, pH variations are narrowly limited in many *in vivo* biological mechanisms, especially targeted neurotransmitter concentration changes due to neuro-activities [17,28,29]. Obviously a RE with less pH dependence is more preferable. Therefore, it is important to understand the pH dependency of the sol-gel-based IrO_x_.

### 3.1. Characterization of Sol-gel-Based pH Electrodes

The two-step sol-gel fabrication process allows producing miniature IrO_x_-based electrodes. Three different electrode dimensions, including 1 mm × 1 mm, 500 μm × 500 μm and 100 μm × 100 μm, were designed. Four electrodes of each type were fabricated and calibrated in order to demonstrate the reproducibility of the fabrication processes. Open-circuit potentials of the IrO_x_ micro-electrodes were measured *versus* a standard Ag/AgCl RE (Basi, West Lafayette, IN, USA) in different standard buffer solutions pH = 4, 7 and 10 (Mettler Toledo, Columbus, OH, USA). The open-circuit potential (*E*) of IrO_x_ electrode in the half-reaction (1) is governed by the Nernst Equation (2):

(1) $$2{\text{IrO}}_{2}+2{\text{H}}^{+}+2{\text{e}}^{-}↔{\text{Ir}}_{2}{\text{O}}_{3}+{\text{H}}_{2}\text{O}$$

(2) $$\text{E}={\text{E}}_{0}+\frac{\text{RT}}{\text{nF}}\text{log}\left({\text{C}}_{{\text{H}}^{+}}{|}_{\text{x}=0}\right)={\text{E}}_{0}-0.059\text{pH}{|}_{\text{x}=0}$$

where *E*_0_ is the standard electrode potential at 25 °C, *R* is a gas constant, and *T*, *F* are denoted for temperature and Faraday's constant. The number of electron exchanged per proton *n* equals to one according to Equation (1). Equation (2) shows that the open-circuit potential depends on the concentration of proton H^+^ near the surface of the electrode C_H_^+^|_x=0_ or pH|_x=0_.

**Figure 4.**
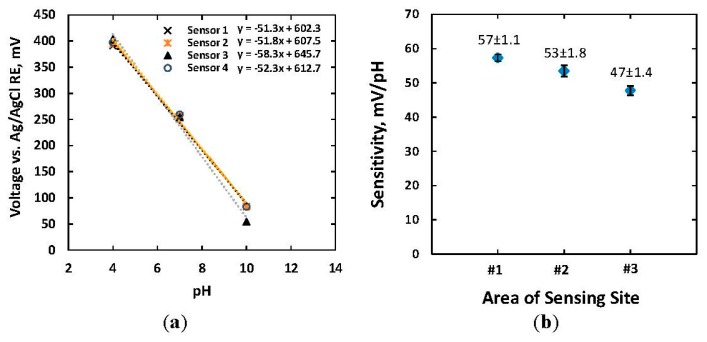
(**a**) The pH sensitivity of four IrO_x_-based electrodes with an electrode size of 500 μm × 500 μm *versus* a standard Ag/AgCl RE; (**b**) Sensitivities of IrO_x_-based electrodes with sizes of (#1) 1 mm × 1 mm, (#2) 500 μm × 500 μm, and (#3) 100 μm × 100 μm were 57 ± 1.1, 53 ± 1.8 and 47 ± 1.4 mV/pH, respectively.

Figure 4a shows the responses of four IrO_x_ electrodes, each of 500 μm × 500 μm size, to standard buffer solutions. The absolute slopes of pH responses were in a range of 51.3–58.3 mV/pH with a linear regression of approximately 0.993, which indicated a Nernst response according to Equation (2). Standard error of the means (SEMs) of the measurements were 5.0 mV, 1.4 mV and 6.9 mV when measuring with commercial buffer solutions of pH = 4, 7, and 10, respectively. Less SEM in the case of the solution pH = 7 indicated higher reproducibility of pH measurements in neutral solutions. The measured pH sensitivities of IrO_x_-based electrodes with various dimensions are shown in Figure 4b. Four electrodes of the same size were measured to obtain statistical variations. There were slight differences in sensitivity for electrodes size of 1 mm × 1 mm and 500 μm × 500 μm, which were 57 ± 1.1 and 53 ± 1.8 mV/pH. The smaller electrodes with a size of 100 μm × 100 μm exhibited less pH dependence of 47 ± 1.4 mV/pH. The relationship between electrode potential and electrode size has not been well-studied yet the Nernst equation does not regard the sensor size. However, the empirical study showed that the smaller electrode possessed less sensitivity. There are two possible reasons for the aforementioned phenomenon. Firstly, the reduced sensitivity of the miniature electrode might relate to the double layer region and diffusion layer formed around the finite space of the microelectrode in electrolyte solutions [35]. The ratio between the thickness of the diffusion layer and physical dimension of the electrode in such case became larger [35]. Bard *et al.* demonstrated that the profile of steady-state ion concentration was proportional to the inverse of distance from the electrode surface [35]. In other words, the local ion concentration and distribution of proton H^+^ near the surface of the electrodes pH|_x=0_ became complex and was adjusted from bulk ion concentration. Thus, the microelectrode showed less sensitivity to pH change of the solution. Secondly, a higher impedance in the smaller microelectrode (∼MΩ) which was comparable to the input impedance of the measurement system reduced the measured voltage from the actual open-circuit potential. An ultra-high input impedance will be needed in such cases.

With a pH sensitivity of 47–57 mV/pH for our miniature-dimension electrodes, the changes of pH in a typical biological environment within a range of 7.0–7.4 corresponded to a maximum variation of around 20 mV. Therefore, the variation in the open-circuit electrode potentials due to pH changes will be negligible compared to the applied bias voltage of 0.7 V in dopamine measurements.

### 3.2. Long-Term Stability of the IrO_x_ Electrodes

**Figure 5.**
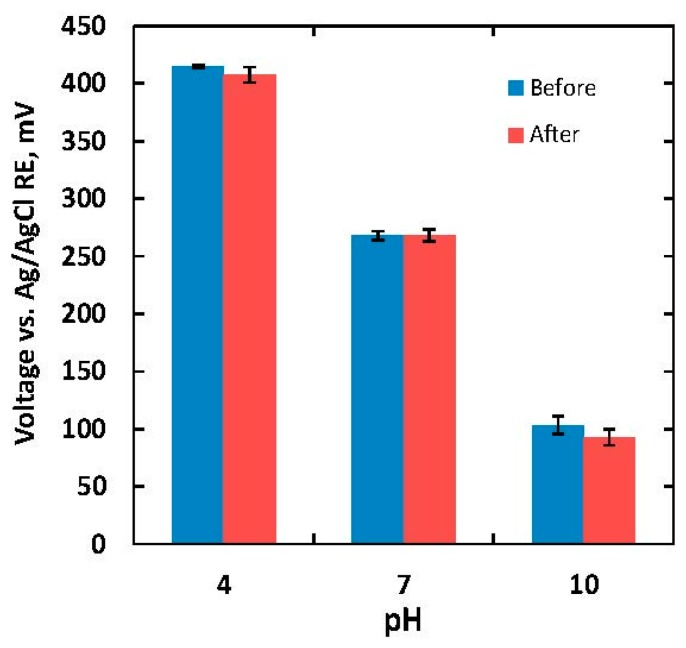
Open circuit potential of a 500 μm × 500 μm IrO_x_ electrode before and after immersing it in 0.05-M PBS solution for 36 h. The sensitivities were 51.8 and 52.4 mV/pH, respectively, before and after the experiment.

Longevity of the reference electrodes is critical in long-term electrochemical recording. Since we target biomedical applications, open-circuit potentials of the IrO_x_ electrode versus an Ag/AgCl RE were examined in two different tests. First, the sensor was tested in a scenario in which the electrode surface first encountered a new biological environment during implantation. The pH sensitivity of the IrO_x_ electrode was characterized when immersed in a 0.05-M phosphate buffered solution (PBS) for 36 h. The PBS solution was chosen since it is a commonly used isotonic solution with physiological pH. An IrO_x_ electrode with a size of 500 μm × 500 μm was fabricated. Figure 5 shows potential responses of the IrO_x_ electrode with different buffer solutions before and after immersing the electrode in the PBS solution.

The measurements were repeated three times. The error bars in Figure 5 indicated the measurement variations. The measured sensitivities before and after the 36-hour experiment were 51.8 and 52.4 mV/pH, respectively. The longevity was then further characterized to verify the performance of sol-gel-based IrO_x−_ electrodes in long-term implantable experiment. The sensors were immersed in different solutions including hydrochloric acid, fetal bovine serum (FBS), and an alkaline buffer solution. The choice of hydrochloric acid was to demonstrate that the sensor could function in a highly acidic biological environment such as inside the stomach with pH of around 2. The FBS solution was chosen because it is commonly used *in vitro* as serum-supplement for cell culture media. Another purpose for the experiment with FBS was to examine how the biofouling effect would affect performance of the sensing film. The alkaline buffer solution of pH = 10 (Mettler Toledo), consisting of sodium hydroxide, potassium chloride and boric acid, was chosen to investigate if the film would be affected in an alkaline environment, and if abundant sodium and potassium ions, which are the most common ions in the biological environment, would affect the sensor lifetime.

**Figure 6.**
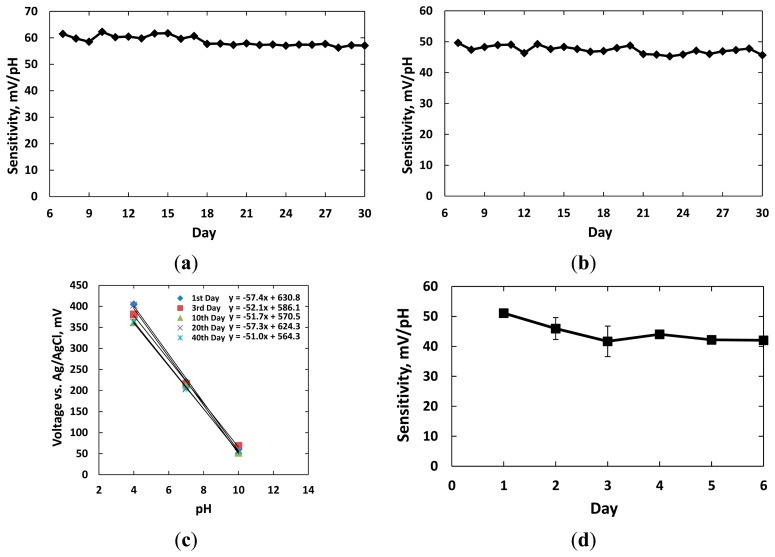
Longevity of IrO_x_-based electrodes was examined by soaking the electrodes in different solutions for a period of time, and keeping in dry condition. Twelve electrodes were classified into four groups. The sensor sensitivities of each group kept in: (**a**) hydrochloric acid solution (pH = 2) for 30 days; (**b**) alkaline solution (pH = 10) containing high concentration of sodium and potassium ions for 30 days; (**c**) standard tissue culture media supplemented with 10% FBS for 40 days; (**d**) dry condition for 6 days, after fabrication were measured.

Twelve sensors with dimension of 1 mm × 1 mm were classified into four groups for the experiments. Three groups were kept in the individual beakers with hydrochloric acid (controlled at pH = 2), alkaline buffer solution (pH = 10), and standard tissue culture media RPMI 1640 supplemented with 10% FBS (Fisher Scientific, Pittsburgh, PA, USA). The electrodes in the fourth group were kept dry in air for comparison. The sensors of each group were tested daily with standard buffer solutions pH = 4, 7, 10 to find the sensitivity curves. After the tests, the sensors were replaced in their original setups. The experiments were carried out for 30 days. Figure 6 shows the sensitivity results in the four different environments.

Figure 6a shows the responses of three sensors kept in the acidic solution with pH = 2 over 30 days. The measurements were conducted after the 6th day. The pH sensitivities of three sensors remained at 57.3 ± 0.6 mV/pH after the 30th day. Standard deviation of the calibration data over 30 days was less than 1.9 mV/pH, which reflected stable performance of these sensors. Figure 6b shows the responses of three sensors kept in the alkaline solution with pH = 10. We noticed that the SU-8 insulation layer visually degraded and wrinkled while soaked in the alkaline solution after five days; however, the experiments were continued. The sensitivities of three sensors in this group were recorded at 48.1 ± 0.55, 47.7 ± 0.61 and 48.0 ± 0.66 mV/pH after 30 days. The reduction in sensitivities was probably due to residues of alkaline solution and hydroxyl ions deeply absorbed and retained at the micro- and nanoscale IrO_x_ pores [8]. The ion exchanges decreased diffusion processes and also altered equilibrium points from the double layer region when new solutions were tested. Both effects changed the electrode potentials of the sensors.

Figure 6c shows the sensitivities of three sensors kept in the FBS solution for 40 days. The average measured sensitivities were 57.4 ± 0.3, 52.1 ± 0.6, 51.7 ± 0.9, 57.3 ± 0.6, and 51.0 ± 0.5 mV/pH on the 1st, 3rd, 10th, 20th and 40th days, respectively. Experiment data showed that the pH responses of these three sensors to the buffer solution pH = 7 decreased 17 mV over 40 days. This change in electrode potentials of the sol-gel IrO_x_ electrodes were negligible compared to the applied bias voltage for amperometric sensors. Therefore, it is promising to use them as pseudo-reference electrodes in biological applications.

We noticed the pH sensitivity was reduced and remained at 42.0 ± 0.6 mV/pH when the sensor were kept dry within 6 days of fabrication, as shown in Figure 6d. The sensor sensitivity recovered to the Nernst performance after immersing in an aqueous solution for three hours. The highest SEM in three measurements of 3.65 mV/pH was higher than the ones for the other electrodes in solutions. The lower sensitivity and higher variations in responses were probably due to dehydration and air filling up the micro- and nanoscale pores in the IrO_x_ film surface. In other words, longer time was needed for the equilibrium points of the redox reaction to reach after ions exchange between solution and surface particles. Therefore, we concluded that the IrO_x_-based electrodes should be preserved or presoaked in a hydrated condition such as PBS solution after fabrication in order to provide stable potentials. Identical conditions are also suggested for most commercially available reference electrodes.

### 3.3. IrO_x_-Based Pseudo-Reference Electrodes

Dopamine is one of the most ubiquitous neurotransmitters of the mammalian central nervous systems (CNS). Recording of dopamine concentration in the CNS could help doctors to understand many diseases such as Parkinson's disease, Alzheimer's diseases and neurodegenerative diseases [4,9,10]. Amperometry and cyclic voltammetry methods have been commonly used to detect dopamine [37,38]. While the amperometry method utilizes a fixed bias voltage, the cyclic voltammetry method sweeps the bias voltage. The applied voltages initiate redox reaction following Equation (3). If we denote dopamine (C_8_H_11_NO_2_) as DA and C_8_H_11_N(OH)_2_ as DAH_2_, then Equation (3) is reduced to Equation (4). Current measurement recorded by amperometry and cyclic voltammetry methods follow the Butler-Volmer Equation (5) [35,37]:

(3) $${\text{C}}_{8}{\text{H}}_{11}{\text{NO}}_{2}+2{\text{H}}^{+}+2{\text{e}}^{-}↔{\text{C}}_{8}{\text{H}}_{11}\text{N}{\left(\text{OH}\right)}_{2}$$

(4) $$\text{DA}+2{\text{H}}^{+}+2{\text{e}}^{-}↔{\text{DAH}}_{2}$$

(5) $$\text{i}={\text{FAk}}_{0}[{\text{C}}_{\text{DA}}\left(0,\text{t}\right){\text{e}}^{-\alpha \text{f}\left(\text{E}-{\text{E}}_{0}\right)}-{\text{C}}_{{\text{DAH}}_{2}}(0,\text{t}){\text{e}}^{-\left(1-\alpha \right)\text{f}\left(\text{E}-{\text{E}}_{0}\right)}]$$

where *F* is the Faraday constant, *A* is active surface area of the electrode, *k_0_* is the rate of the reaction at equilibrium state, *C_x_*(0,*t*) is the concentration of chemical *x*, and *α* is transfer coefficient. Constant *f* is calculated as *F/RT*, where *R* is the gas constant and *T* is temperature. According to Equation (5), the redox current *i* depends on the concentrations of DA and DAH_2_ as well as the bias voltage *E* between the WE and RE. In our experiment, we implemented the amperometric method to detect the concentration of DA. The amperometric sensor consisted of a working (WE) and a reference (RE) electrodes. Two DA sensors were fabricated. They both employed gold electrodes with a size of 50 μm × 100 μm as the working electrodes. However, one sensor referred to a micro IrO_x_ pseudo-RE while the other one was measured with a separated standard glass-tube Ag/AgCl RE. The IrO_x_ pseudo-RE also had a size of 50 μm × 100 μm. The glass-tube Ag/AgCl RE consisted a Ag/AgCl wire with a diameter of 500 μm and a length of 5 mm. A fixed bias voltage of 0.7 V was applied between the RE and the gold WE for each DA sensor. The 0.7-V bias voltage was chosen since it generated peak redox currents [37,38]. Two sensors were kept in a beaker containing 40 mL of 0.05-M PBS solution. The temperature of the solution was maintained at 37 °C with a water bath. The concentration of DA in the PBS solution was raised by incremental addition of 2.5 μM of DA (Sigma-Aldrich, St. Louis, MO, USA) under constant stirring by a magnetic rod. Electrical current was recorded with a commercial potentiostat (Pinnacle, Inc., Lawrence, KS, USA).

**Figure 7.**
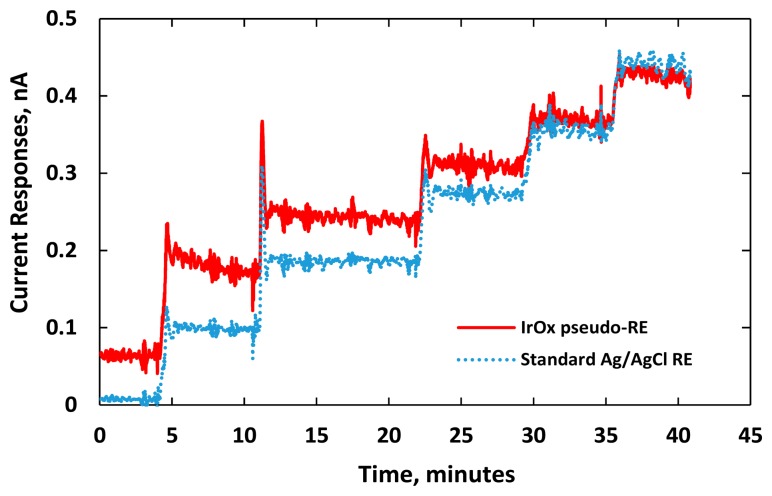
Electrical current responses of two DA sensors for incremental additions of 2.5-μM dopamine to 40 mL of 0.05-M PBS solution. Both sensors had identical gold WEs but with different REs: (red) the planar IrO_x_ pseudo-RE; and (blue) a standard glass-tube Ag/AgCl RE.

Figure 7 shows distinct responses to each increment of DA concentration. We noticed electrical current overshoots on both sensors as soon as the DA was added. The phenomenon happened in the transient period due to artifact noises or a local rise of ion concentration around the electrode [3,35,39]. The transient time between the adding of DA and the stable current response was short for both sensors demonstrating fast sensing electrode performance. Noises recorded by both sensors were also similar. The current responses damped down and reached their steady state after one minute.

**Figure 8.**
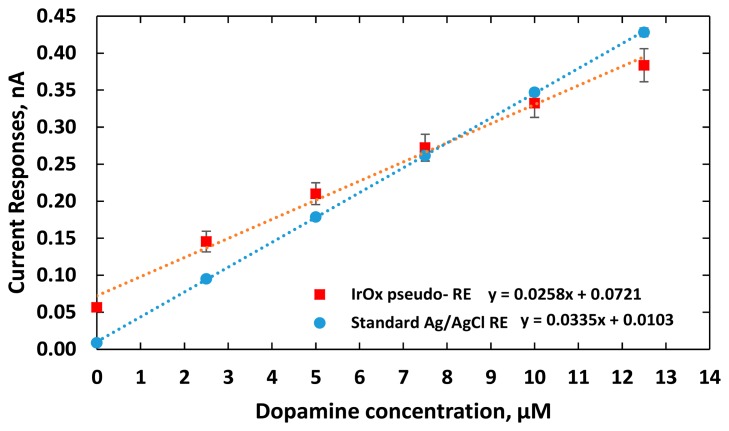
The sensitivity (slope) and repeatability (indicated by the standard error of the mean, SEM) of two DA sensors after three trials. Both sensors have gold working electrodes with a size of 50 μm ×100 μm. One sensor has an external IrO_x_ pseudo-RE (red line). The other sensor used an external standard Ag/AgCl reference probe (blue line).

The experiments were repeated three times recording electrical currents at various DA concentrations. As shown in Figure 8, the sensitivities of the two DA sensors were measured as 25.8 ± 0.07 and 33.5 ± 0.01 pA/μM, for using the IrO_x_ pseudo-RE and a standard Ag/AgCl RE, respectively. Due to the fact that the highly electroactive IrO_x_ has fast reversible redox reactions or a larger rate of reaction *k_0_* [40,41], the DA sensor using the IrO_x_ pseudo-RE has an output with higher baseline current. The measurements also indicated that the sensor exhibited less sensitivity than the one with a standard reference probe. It should be noted that the pseudo-RE has a miniature size (50 μm × 100 μm). According to Equation (5), its sensitivity is expected to be less due to its higher impedance. Besides the electrode dimensions, to optimize the sensitivity, other factors should also be considered including spacing between the WE and RE, acquisition instrumentation configurations (input impedance and gain) and working electrode materials.

### 3.4. IrOx-Based Working Electrodes

We have also investigated the possibility of implementing IrO_x_ electrodes for both working and reference electrodes. Dopamine sensors with the WE and RE on the same probe were fabricated with the same sol-gel batch processes. Each electrode had a size of 50 μm × 100 μm. A bias voltage of 0.7 V was applied between the two electrodes. The tests were carried out by incrementally adding DA to the PBS solution, as before. At the same time, another DA sensor consisting of a gold WE and a standard Ag/AgCl RE was placed in the beaker to compare results.

**Figure 9.**
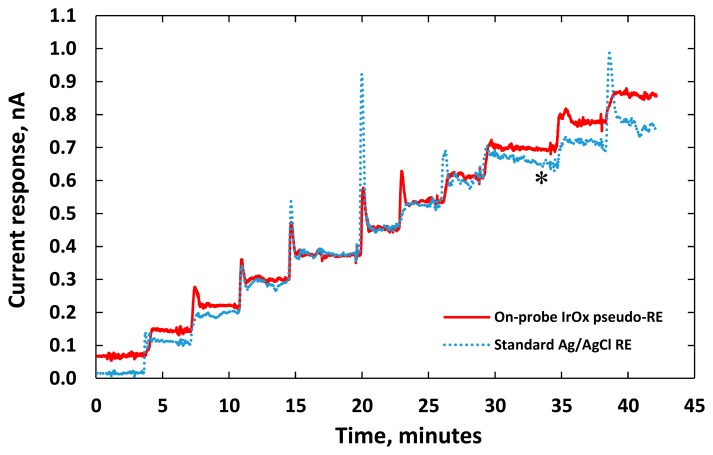
Current responses of two DA sensors for incremental additions of 2.5 μM dopamine to 40 mL of 0.05-M PBS solution: (red) the integrated DA sensor had an IrO_x_ WE and an IrO_x_ pseudo-RE on the same probe; and (blue) the other DA sensor had a gold WE with an external standard AgCl/Ag RE probe.

Figure 9 shows electric current responses of these two DA sensors while incremental adding 2.5-μM DA to raise the concentration of the PBS solution. The performance of the DA sensor with integrated IrO_x_ WE and RE is similar to the one with a gold WE and an external Ag/AgCl reference probe. When the concentration of DA increased above 30 μM, the current responses of the DA sensor with the gold WE degraded. The degraded performance (blue curve) is indicated by * in Figure 9. The degradation commonly happened with micro-electrodes due to accumulation of the byproduct (DAH_2_) generated by the redox reaction within the micro-scale sensing area [9,42]. This issue became more severe with higher background currents causing additional surface oxide or adsorbed hydrogen layer on the electrode surface [43,44]. In the case of using sol-gel IrO_x_ as the WE (red curve), the degradation was not observed. The IrO_x_ sol-gel matrices with high porosity significantly improved the surface area of the micro-electrodes [44,45]. The degradation with the IrO_x_ WE was only initiated with a higher redox current. Therefore, the IrO_x_-based WE could have higher dynamic range compared to the gold WE.

**Figure 10.**
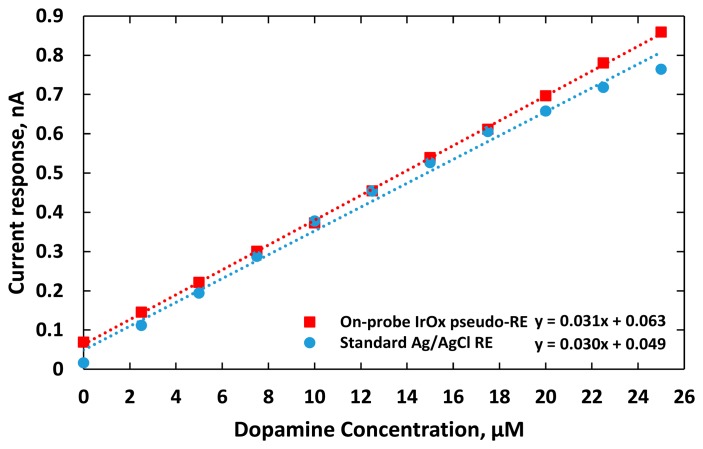
Sensitivities of (red) the DA sensor with integrated IrO_x_ WE and IrO_x_ pseudo-RE on the same probe; and (blue) the DA sensor that employed a gold WE and an external commercial glass-tube Ag/AgCl RE probe.

The electric current responses of the sensors were plotted as a function of DA concentration as shown in Figure 10. The DA sensor utilizing IrO_x_ WE and RE had a higher linear regression of 0.9997. It also coupled with less noise although it still had a higher baseline, compared to the one using the external Ag/AgCl RE probe. This is due to the fact that the distance between the IrO_x_ WE and RE was only 500 μm, which was much shorter than the distance between the gold WE and the external Ag/AgCl RE probe that had a fixed distance of 2 cm in our *in vitro* setup. Consequently, additional interferences from dynamic ion distribution between the electrodes with greater separation in solution added more noises into recorded potentials. The average standard deviation of measurement reflecting the dispersion of the measurement from the average sensor responses at different DA concentrations was 6 pA with the DA sensor integrating the IrO_x_ WE and RE, while for the one using the standard Ag/AgCl RE was around 9 pA. The sensitivities of these two electrodes were 31.7 and 30.3 pA/μM, respectively. The limits of detections (LOD), the lowest quantity of dopamine that can be distinguished [46], were calculated to be 0.01 and 0.5 μM, respectively. Re-calibration after six days manifested the sensor sensitivities with a standard deviation of 27.8 ± 2.9 pA/μM and 28.6 ± 4.0 pA/μM, respectively.

## 4. Conclusions

The sol-gel fabrication process utilizing a two-step heat treatment was developed to fabricate micro-scale IrO_x_ electrodes on a flexible polymeric substrate. The fabrication processes allowed easy removal of the sacrificial mask layer after patterning while maintaining the film quality. The properties of the IrO_x_ films were similar to the ones fabricated by the one-step heat treatment processes. Sensitivity to pH and longevity of the microelectrodes were studied. Sensor electrode performance did not change significantly over 30 days when they were kept in different environments designed to demonstrate their use in biomedical applications. Electrodes were demonstrated for application in DA concentration recording. The DA sensor with sol-gel IrO_x_ working and pseudo-reference electrodes on the same probe had similar sensitivity but less noise and better limit of detection than the one using a gold working electrode and an external Ag/AgCl reference probe. The flexible sensor with arrayed and integrated IrO_x_ electrodes can be utilized in long-term implants to detect *in situ* multiple neurotransmitters for diagnosis of neural disorders.
